# Occupational Injury and Its Correlated Factors among Small-Scale Industry Workers in Towns of Bale Zone, Southeast Ethiopia

**DOI:** 10.1155/2019/4987974

**Published:** 2019-12-27

**Authors:** Nagasa Dida, Jiregna Darega, Feyisa Lemesa, Jeylan Kassim, Bedasa Woldemichael

**Affiliations:** ^1^Department of Public Health, Medicine and Health Science College, Ambo University, P.O. B. 19, Ambo, Ethiopia; ^2^Department of Nursing, Goba Referral Hospital, Madda Walabu University, P.O. B. 302, Bale-Goba, Ethiopia; ^3^Department of Public Health, Goba Referral Hospital, Madda Walabu University, P.O. B. 302, Bale-Goba, Ethiopia; ^4^Department of Nursing, College of Health Science, Arsi University, P.O. B. 396, Asella, Ethiopia

## Abstract

**Introduction:**

In developing countries, the laborer forces have managed many of the industrial works. As a result, the process of the work has put the health and lives of workers at risk. Thus, this study was designed to assess occupational injury and its correlated factors among small-scale industry workers in the towns of Bale Zone, Southeast Ethiopia.

**Methods:**

An institution-based cross-sectional study design was employed among five hundred ninety small-scale industries in towns of Bale zone, Southeast Ethiopia, in March to April 2016. Multistage sampling was applied to recruit the study subjects. Data were collected through interviewer-administered questionnaires. A structured questionnaire addressing the objectives of the study was used. EpiData was used for data entry, and the data were exported to SPSS windows version 20 for analysis. Descriptive statistics like frequency and percentage were used for the prevalence, whereas binary and multiple logistic regressions were employed to identify the predictors of the outcome variable.

**Results:**

A total of 574 workers from different small-scale industries, including woodwork, metalwork, and concrete block construction, participated in the study with a response rate of 97.3%. In this study, among the total participants of the study, 43.2% (248), 30% (172), and 21.6% (124) of them had encountered lifetime, last one year, and six months occupational injury, respectively. Taking health and safety training, presence of any things on the floor that can cause accidents and occupational risk perception were independent predictors of occupational injury. The presence of any things on the floor that can cause accidents and having a low-risk perception increases occupational injury by 12.69 [AOR: 12.69, (1.67–96.13)] and 2.84 [AOR: 2.84, (1.80–4.49)], respectively.

**Conclusion:**

About one in three occupational injuries occurred among small-scale industry workers. Health and safety training should be provided for every worker in small-scale industries. Supportive supervision focusing awareness creation, economic stability, and health care from health office, social and labor affair office, and other concerned body is highly recommended. District or town health office should address the identified factors to promote the health of the workers.

## 1. Background

Occupational injuries are physical harm occurring due to work performed at a given company and causing a functional alteration and/or bodily lesion in the worker [1]. Today, it is one of the major public health problems worldwide. Globally, 271 million people encountered work-related injuries of which two million of them die annually [2]. In developing countries, usage of laborer forces in the industrial work process puts the health and lives of workers at risk [3], which makes 10 to 20 times higher occurrences of the injuries as compared to developed countries [4]. More than 90% of companies were micro and small-scale enterprises whose working environments are very poor, and workers are often excluded from all legal protection [3]. In Africa, its general informality and poverty of the country result in environmental degradation and low standard of occupational safety [5], which leads to greater frequency and severity of the injury [6].

Occupational injury and diseases that occurred in the wood sector constitute a significant public health problem. It induces high psychological burden and money to families and society; 2.2 million occupational-related death and 270 million occupational injuries occur annually. In 2008, the number of fatalities increased to 116 deaths per 100 000 workers [7, 8]. In a similar manner, construction industry is also categorized under the most hazardous industries where work-related injuries happen both in industrialized and industrializing countries and remains a major public health problem [9]. In Zimbabwe, wood and wood products ranked among the major accidents, injuries, and diseases in industries [10].

Mortality rates that happen due to occupational accident are five to six times higher in many developing countries compared to developed countries. Due to undocumented and poor recording system, the actual figure of occupational injury is not known exactly [4].

Data on occupational health and safety issues are quite limited in the microenterprise sector, and subjective evidence seems to suggest that majority of small-scale industries do not engage in safeguarding practices for the well-being of workers and family members [5]. For evidence-based occupational hazard prevention and related health promotion program, the result of this study has paramount value for policy makers, local, national, and international government, and nongovernment organization. Researchers could also use the finding as a literature and could be a spring board for further research. Thus, the aim of this study was to describe occupational injury and identify its risk factors among small-scale industry workers in the towns of Bale Zone, Southeast Ethiopia.

## 2. Methods

### 2.1. Study Setting and Participation

Institutional-based cross-sectional study design was conducted among workers of wood/metalwork and concrete construction in the Towns of Bale Zone from March to April, 2016. Bale zone is found in Oromia Regional State at 430 km away from Addis Ababa, the capital city of Ethiopia to the Southeast. It has three administrative towns and 18 district towns. The total population of the zone was 1,708,910. Bale zone had a total of 8, 259 small-scale industries with a total of 51,362 workers (male: 30,911, female: 20,451) of which 361 of them were woodwork, metalwork, and concrete and construction having 5415 (male: 5087; female: 328) workers [11].

The sample size was determined using single population proportion formula (Fisher's formula) with the assumption of level of confidence 95%, sampling error tolerated 5% and 58.2% was a proportion of one year occupational injury, which was taken from a study done in Mekele city [12], 5% nonresponse rate, and 1.5 of design effect were considered, and the final sample size became 590.

**Figure 1:**
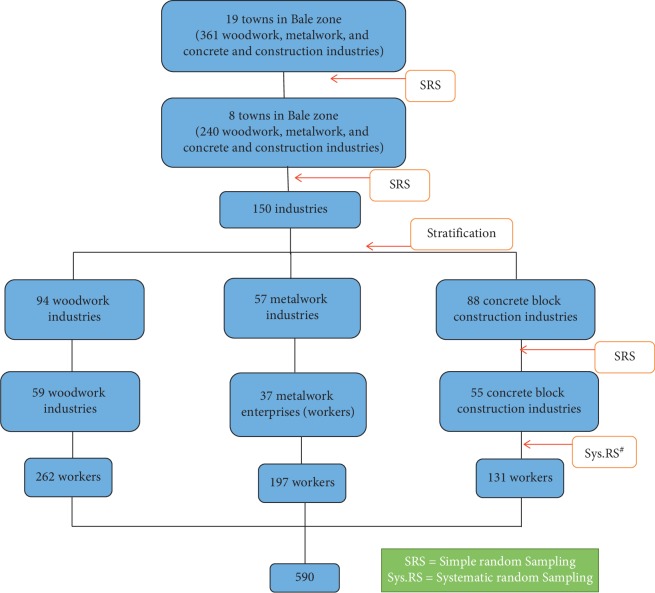
Sampling procedure for occupational injury and its correlated factors among small-scale industry workers in Towns of Bale Zone, Southeast Ethiopia, 2016.

From 19 towns of the zone, eight towns namely Dallo Manna, Gollolcha, Robe, Goba, Ginnir, Agarfa, Goro, and Gasara were selected randomly. There were a total of 240 small-scale industries/enterprises (woodwork = 94, metalwork = 57, and concrete block construction = 89) in these eight towns. The small-scale industries/enterprises were stratified into woodwork, metalwork, and concrete construction based on the nature of the work. Of the total 240 small-scale industries, 150 of them were selected randomly. Probability proportion sample was used to determine small-scale industries to be in the study from the selected towns and for the strata too. Number of workers in the industries was surveyed before the study, and the study participants were allocated proportionally. To address the study subject, all workers were included in case the industry had three and above workers, and every other individuals were selected if there were four and above workers (Figure 1).

### 2.2. Data Processing and Management

#### 2.2.1. Questionnaire and Variables

Structured questionnaire and observational checklist covering occupational injury and related variables were adapted from pertinent literatures [1, 4, 12–14]. The questionnaire encompasses the main variables like sociodemographic characteristics, health and safety practices at the workplaces, occupational injury and workers' health behaviours, or habits. Data were collected through interviewer administered questionnaire and observation checklist.

The questionnaire was translated into the local language (Afaan Oromo and Amharic) and retranslated back to English to check its consistency by language experts. Data collectors and supervisors were trained on the objective of the study and data collection process for two days. Pretest was done on 5% of similar population in unselected town before the actual study. On top of this, the investigators checked for the collected data on daily bases.

#### 2.2.2. Statistical Analysis

Data were entered using EpiData and exported to SPSS windows version 20.0 for analysis. Both descriptive and analytical analyses were done. The descriptive results have been presented by tables and graphs. Bivariate logistic regression analysis was used to identify associations between independent and study variables. The possible effects of confounders were controlled through multivariate logistic regression analysis. The association between the explanatory and dependent variables were assessed at *p* value of 0.05.

## 3. Results

### 3.1. Sociodemographic Characteristics

A total of 574 small-scale industry workers, namely, woodwork, metalwork, and concrete block construction, participated in the study with a response rate of 97.3%. Sixty percent of the organizations were owned privately, whereas the remaining 40% were micro and small-scale industries. The mean age of the respondents was 25.43 (standard deviation (SD) ± 7.15). The majority 95.5% (548) of the respondents were male. Sixty-nine percent (398) of the respondents were Orthodox Christian by religion followed by Muslim. Regarding the marital status of the respondents, 60.3% (346) of them were single and 1% (9) of them were divorced. Forty-nine percent (282) of the respondents had the educational status of secondary school, and 20% of them had a certificate and above (Table 1).

### 3.2. Health and Safety Practices at the Workplaces

The median of work experience of the study participants was 3 years (SD ± 4.84). Of the total study participants, only 12.5% (72) of them had college training before joining their work while the rest (87.3%) of the respondents started the work through experiences (Table 2).

### 3.3. Occupational Injury and Related Factors

**Figure 2:**
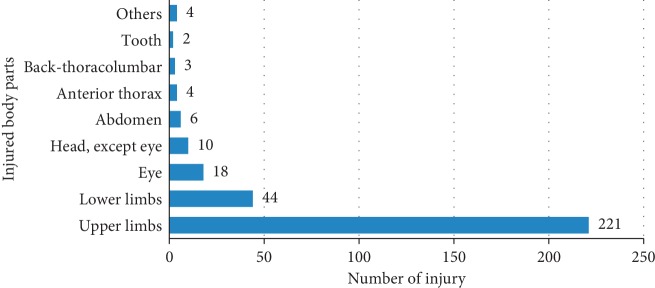
Body parts injured among small-scale industry workers in towns of Bale Zone, Southeast Ethiopia, March to April 2016 (*n* = 248; Multiple body part injury was counted in a single case).

Of the total 574 study participants, 43.2% (248) of them had encountered lifetime occupational injury. Specifically, 30% (172), 21.6% (124), and 10.3% (59) of injury occurred in the last one year, six months, and two weeks, respectively, prior to the study. Regarding the frequency of injury, 58.9% (146) of the respondents injured once, and the rest 41.1% (102) injured two and above times. Among those encountered injuries in their life, 19.4% (48) of them faced severe injury. From the total injury, 89.1% (221), 17.7% (44), and 7.3% (18) of them happened to upper limbs, lower limbs, and eye, respectively (Figure 2).

**Figure 3:**
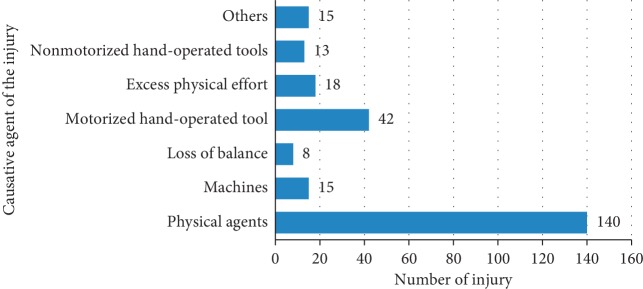
Causative agents of injury among small-scale industry workers in towns of Bale Zone, Southeast Ethiopia, March to April 2016 (*n* = 248; Multiple causative agent was counted in a single case).

Because of occupational injury, nearly twenty percent of the study participants were absent from their regular work of which 12.2% of them absent for one to two weeks and the rest eight percent absent for more than two weeks. Eight individuals had a history of hospital admission. Physical agents were the major causes of injury among respondents who faced injury in their life (Figure 3).

Occupational injury and workers' health behaviours or habits of the total study participants, 20% (115) of them had history of drinking alcohol. Similarly, 24.2% (139) of the study participant used khat (*Catha edulis*). There was also sleeping disorder among 15.2% (87) of the respondents, and similar figures (14.6%) of the respondents had medical problems. More than forty percent of the respondents work at night using electric light (Table 3).

### 3.4. Factors Associated with One-Year Occupational Injury

To identify associated factors of occupational injury among small-scale industry workers in the towns of Bale zone, bivariate logistic regression and multivariate logistic regression were applied at a *p* value less than 0.05.

Using bivariate logistic regression, a type of small-scale industry, supervision from any organization health office and other concerned office, having health and safety training, alcohol use, working at night, presence of fallen things on the floor that can cause accidents, use of personnel protective equipment, and occupational injury risk perception had statistically significant association.

Those respondents working in small-scale industry of metalwork and concrete block construction were at lower risk of getting an occupational injury compared to those respondents working in metalwood work industry with an odd of 0.5 and 0.6, respectively. Those small-scale industry (SSI) workers who had no supervision from health office encountered injury two times of their counterpart [COR: 1.83 (1.18–2.83)]. Similarly, those workers engaged in working at night and use alcohol drinking were 2.06 [COR: 2.06 (1.43–2.96)] and 1.76 [COR: 1.76 (1.15–2.70)] times at risk of occupational injury compared to their counterparts. Presence of any things on the floor that can cause accidents to extremely increase occupational injury by eleven times compared to those workers doing in small-scale industry without things causing accidents on the floor [COR: 10.98 (1.47–82.04)]. Those respondents with low risk perception of occupational injury were also 3.5 times more likely to encounter occupational injury compared to those workers with high risk perception of occupational injury [COR: 3.52 (2.36–5.25)]. However, those workers who took health and safety training and using personnel protective equipment were 0.56 and 0.64, respectively, times less likely to be injured by their professional-related activity [COR: 0.56 (0.32–0.98) and COR: 0.64 (0.44–0.92)] (Table 4).

When factors associated with occupational injury were adjusted for confounding factors, absence of supervision from health office, taking health and safety training, and presence of any things on the floor that can cause accidents and occupational risk perception became independent predictors of occupational injury. Those workers who took health and safety training in their profession were 67% times less likely to be injured compared to those workers who did not take the training [AOR: 0.33, (0.15–0.73)]. Presence of any things on the floor that can cause an accident and having a low risk perception increase occupational injury by 12.69 [AOR: 12.69, (1.67–96.13)] and 2.84 times 2.84 [AOR: 2.84, (1.80–4.49)], respectively (Table 4).

## 4. Discussion

This study assessed the prevalence and associated factors of occupational injury among small-scale industries' workers in the towns of Bale Zone, Oromia Regional State, Southeast Ethiopia. Besides, the occupational risk perception of the workers was identified using variables, which best explains the risk perception. The relation to the lifetime, one year, six months, and two weeks prevalence of occupational injury among small-scale industry workers were 43.2%, 30%, 21.6%, and 10.3% respectively.

One year occupational injury among small-scale industry workers in the towns of Bale zone was 30%. This result is lower by half than the one year occupational injury among similar workers in Mekele City, which was 58.2% [12]. In the Amhara region, Gondar zone, and Bahir Dar city, similar figures of injuries were reported [14, 15]. Additionally, annual work-related injuries in the Gondar city among building construction workers were 38.7%, which was a little bit higher compared to this study [13]. But in Gabon, nearly similar figure (24.2%) of woodworkers encountered occupational injury in 2007 [16].

High proportion, 89.1% (221), of injury occurred to upper limbs followed by lower limbs, 17.7% (44). Also in Mekele city, high proportion of injury was upper limbs injury followed by the lower limbs with a proportion of 49.4% (218) and 20.6% (91) respectively. With a difference of 10%, proportion of severe injury was lower in this study than that of Mekele city (30.4%) [12]. In Ethiopia (Bahir Dar) and Brazil, fingers and hands, respectively, were the primarily affected body parts of occupational injury [1, 15]. Likewise, in Italy and Pakistan, hand and finger injury were the typical woodworking accidents [17, 18]. With decreasing order, physical agents, motorized hand-operated tools, and excessive physical efforts were the most ultimate causative agents of these injuries that are consistent with the study done in Brazil [1].

The proportion of those respondents who have been using personal protective equipment regularly was 22.1% (127) that is lower compared to small-scale industry workers in Mekele city where 33.8% (256) of them used personal protective equipment properly and consistently [12]. In small-scale metal press industries of Shahdrah Town, Lahore, Pakistan, most of the workers have not worn the personal protective equipment [18].

Using bivariate logistic regression, type of small-scale industry, supervision from any organization including health office, having health and safety training, alcohol use, working at night, and presence of fallen things on the floor that can cause accident, use of personnel protective equipment and occupational injury risk perception had statistically significant association. In line of this study finding, study done in Mekele city identified worker job category, health and safety training, use of personal protective equipment, and working at night were identified as these variables had an association with occupational injury. In both studies, personal protective equipment and training showed preventive effect while doing at night increase the odd of occupational injury [12]. On the contrary, a study done in Gondar among concrete block manufacturing workers identified vocational training increased the odd of occupational injury by 2.4 times [13].

In the multivariate logistic regression, the independent predicator variables identified were absence supervision from health office, taking health and safety training, and presence of any things on the floor that can cause accident and occupational risk perception. Those workers who took health and safety training on their profession were 0.33 times less likely to be injured compared to those workers who did not take the training. Among Mekele city, small-scale industry workers those respondents who did not take the training were 2.41 times at risk of occupational injury [12]. In North Bahir Dar, it was also found to be protective to occupational injury [14]. Low occupational risk perception also increases the odds of occupational injury by 2.84. In woodworking industries of Zimbabwe, much attention has not been given to the safety of processing machines, equipment, tools, and their link to health requirements [19].

As payment of most of the workers was based on materials they produce and/or daily based employment, they might be in fear of their boss, which might affect their concentration of responding to the interview. They were in a hurry to go back to their work. This might affect the quality of data of the study. Recall bias could also be the factor for under the report of occupational injury. The undermining of reporting simple injury was also observed.

## 5. Conclusions and Recommendations

About one in three occupational injuries occurred to small-scale industry workers. Upper and lower limbs were the most affected body parts. Laceration, abrasion, and cut were among the types of injuries. Physical agents, motorized hand-operated tools, excessive use of physical effort, and machine were the major perceived causes of injuries. Health and safety training, the existence of physical agents on the floor capable of causing an accident and occupational risk perception, were the predictors of occupational injury.

Personal protective equipment, especially to the upper and lower limbs, has to be used by the workers. Health and safety training should be provided for every worker of small-scale industries. Supportive supervision focusing awareness creation, economic stability, and health care from health office, social and labor affair office, and other concerned body is highly recommended. Zonal health department, district, or town health offices should address the identified factors to promote the health of workers who engage in such poverty reduction strategy. Further study using strong study design, and occupational health and safety policy implementation are recommended.

## Figures and Tables

**Table 1:** Sociodemographic characteristics of the small-scale industry workers in towns of Bale Zone, Oromia Regional State, Southeast Ethiopia, March to April 2016.

Variables	Frequency (*N* = 574)	Percent (%)
Industry owner		
Private	341	59.4
Enterprise	233	40.6

Type of industries		
Woodwork	253	44.1
Metalwork	114	19.9
Concrete—construction	131	22.8
Wood and metalwork	76	13.2

Sex		
Male	548	95.5
Female	26	4.5

Marital status		
Married	219	38.2
Single	346	60.3
Divorced	9	1.6

Religion		
Orthodox	398	69.3
Muslim	125	21.8
Protestant	45	7.8
Other	6	1.0

Ethnicity		
Oromo	383	66.7
Amhara	159	27.7
Tigre	5	0.9
Other	27	4.7

Educational status		
Unable to read and write	15	2.6
Primary school	162	28.2
Secondary school	282	49.1
Certificate and above	115	20.0

**Table 2:** Health and safety practices at the workplaces among small-scale industry workers in Towns of Bale Zone, Oromia Regional State, Southeast Ethiopia, March to April 2016.

Variables	Frequency (*N* = 574)	Percent (%)
Tea break apart from the lunch		
Yes	228	39.8
No	345	60.2

Rest per week (day)		
No rest	57	9.9
One day	467	81.4
Two or three days	50	8.7

Are you satisfied with your workmate(s)		
Yes	543	94.6
No	31	5.4

Is the work place physical environment satisfactory		
Yes	481	83.8
No	93	16.2

Lack supervision on injury prevention from any organization including health office		
Yes	105	18.3
No	469	81.7

Had health and safety training		
Yes	83	14.5
No	491	85.5

Satisfaction to their job		
Yes	534	93.0
No	40	7.0

Personal protective equipment usage		
Never	179	31.2
Regularly	127	22.1
Occasionally	268	46.7

**Table 3:** Workers' health behaviours or habits among small-scale industry workers in towns of Bale zone, Southeast Ethiopia, March to April 2016.

Variables	Frequency (*N*)	Percent (%)
Drinking alcohol		
Yes	115	20.0
No	459	80.0

Sleeping disorder at night		
Yes	87	15.2
No	487	84.8

Smoke cigarette		
Yes	62	10.8
No	512	89.2

Chew chat		
Yes	139	24.2
No	435	75.8

Work at night		
Yes	236	41.1
No	338	58.9

**Table 4:** Factor associated with occupational injury among small-scale industry workers in towns of Bale Zone Southeast Ethiopia, April to March 2016.

Covariances	Occupational injury		COR (95% CI)	AOR (95% CI)
	Yes	No		
Type of SSI^*∗*^				
Woodwork	180	73	0.59 (0.35–1.00)	1.21 (0.55–2.64)
Metalwork	79	35	0.64 (0.35–0.1.18)	1.07 (0.45–2.52)
Concrete block construction	98	33	0.49 (0.27–0.89)	1.29 (0.55–3.04)
Metal-wood work	45	31	1.00	1.00

Lack supervision from any organization including health office				
Yes	62	43	1.83 (1.18–2.83)	3.15 (1.70–5.81)^*∗*^
No	340	129	1.00	1.00

Took safety training				
Yes	66	17	0.56 (0.32–0.98)	0.33 (0.15–0.73)^*∗*^
No	336	155	1.00	1.00

Use alcohol				
Yes	69	46	1.76 (1.15–2.70)	1.56 (0.93–2.61)
No	333	126	1.00	1.00

Working at night				
Yes	144	92	2.06 (1.43–2.96)	1.39 (0.89–2.17)
No	258	80	1.00	1.00

Any things on the floor that can cause accident				
Yes	282	129	10.98 (1.47–82.04)	12.69 (1.67–96.13)^*∗*^
No	24	1	1.00	1.00

Use PPE				
Yes	201	105	0.64 (0.44–0.92)	0.81 (0.51–1.28)
No	201	67	1.00	1.00

Risk perception				
Low	83	173	3.52 (2.36–5.25)	2.84 (1.80–4.49)^*∗∗*^
High	165	153	1.00	1.00

SSI—small-scale industry; PPE—personnel protective equipment; ^*∗*^*p* value <0.05, ^*∗∗*^*p* value < 0.01; COR—crude odds ratio; AOR—adjusted odds ratio.

## Data Availability

The data used to support the findings of this study are available from the corresponding author upon request.
